# Registration of antimicrobials, Kenya, Uganda and United Republic of Tanzania, 2018

**DOI:** 10.2471/BLT.19.249433

**Published:** 2020-06-19

**Authors:** Rosanna Lyus, Allyson Pollock, Moses Ocan, Petra Brhlikova

**Affiliations:** a Centre for Regulatory Science, Population Health Sciences Institute, Baddiley-Clark Building, Newcastle University, Newcastle upon Tyne, NE2 4AX, England.; b Department of Pharmacology & Therapeutics, Makerere University, Kampala, Uganda.

## Abstract

**Objective:**

To determine the proportion of essential and non-essential antimicrobial medicines that are registered on the drug registers in Kenya, Uganda and United Republic of Tanzania.

**Methods:**

We categorized all antimicrobials on the national drug registers and essential medicines lists of the three countries using the British National Formulary. We also categorized all antibiotics according to the World Health Organization access, watch and reserve (AWaRe) classification. We calculated the proportions of essential and non-essential antimicrobials that were registered by antimicrobial class and AWaRe classification.

**Findings:**

In 2018, Kenya had 2105 registered antimicrobials, Uganda had 1563 and the United Republic of Tanzania had 1327. Of these medicines, 1353 (64.3%) were non-essential in Kenya, 798 (51.1%) in Uganda and 706 (53.2%) in the United Republic of Tanzania. Kenya had 160 antimicrobials on its national essential medicines lists, Uganda had 187 and the United Republic of Tanzania had 182; of these, 33 (20.7%), 50 (26.7%) and 52 (28.6%) were not registered, respectively. High proportions of antimycobacterial and antiparasitic medicines were not registered. Of essential access antibiotics, 14.3% (4/28) were not registered in Kenya, 8.6% (3/35) in Uganda and 20.5% (8/39) in the United Republic of Tanzania, nor were 25.0% (3/12) of watch antibiotics in Kenya, 14.3% (2/14) in Uganda and 19.1% (4/21) in the United Republic of Tanzania.

**Conclusion:**

Suboptimal registration of essential antimicrobials and over-registration of non-essential antimicrobials may encourage inappropriate use, especially since non-essential antimicrobials do not appear on national treatment guidelines. Countries should prioritize registration of the antimicrobial medicines on their essential medicines lists.

## Résumé

**Objectif:**

Déterminer la quantité d'antimicrobiens essentiels et non essentiels figurant dans les registres de médicaments au Kenya, en Ouganda et en République-Unie de Tanzanie.

**Méthodes:**

Nous avons classé tous les antimicrobiens inscrits dans les registres nationaux et listes de médicaments essentiels des trois pays en nous basant sur le British National Formulary. Nous avons également catégorisé tous les antibiotiques conformément à la classification AWaRe (Access, Watch et Reserve) mise en place par l'Organisation mondiale de la Santé. Nous avons calculé les proportions d'antimicrobiens essentiels et non essentiels répertoriés en fonction de la catégorie d'antimicrobiens et de la classification AWaRe.

**Résultats:**

En 2018, le Kenya comptait 2105 antimicrobiens enregistrés, l'Ouganda en avait 1563 et la République-Unie de Tanzanie, 1327. Parmi ces médicaments, 1353 (64,3%) étaient non essentiels au Kenya, 798 (51,1%) en Ouganda et 706 (53,2%) en République-Unie de Tanzanie. Le Kenya possédait 160 antimicrobiens sur ses listes nationales de médicaments essentiels, l'Ouganda en dénombrait 187 et la République-Unie de Tanzanie en recensait 182; respectivement 33 (20,7%), 50 (26,7%) et 52 (28,6%) n'étaient pas répertoriés. Une quantité élevée de médicaments antimycobactériens et antiparasitaires ne figuraient nulle part. Parmi les antibiotiques essentiels de la catégorie Access, 14,3% (4/28) n'étaient pas référencés au Kenya, 8,6% (3/35) en Ouganda et 20,5% (8/39) en République-Unie de Tanzanie; il en allait de même pour 25,0% (3/12) des antibiotiques de la catégorie Watch au Kenya, 14,3% (2/14) en Ouganda et 19,1% (4/21) en République-Unie de Tanzanie.

**Conclusion:**

L'enregistrement insuffisant des antimicrobiens essentiels d'une part et, de l'autre, l'enregistrement excessif des antimicrobiens non essentiels pourraient favoriser un usage inapproprié, d'autant plus que les antimicrobiens non essentiels ne sont pas mentionnés dans les directives de traitement nationales. Les pays devraient privilégier l'enregistrement des antimicrobiens sur leurs listes de médicaments essentiels.

## Resumen

**Objetivo:**

Determinar el porcentaje de los medicamentos antimicrobianos esenciales y no esenciales que están inscritos en los registros de medicamentos de Kenya, la República Unida de Tanzania y Uganda.

**Métodos:**

Se clasificaron todos los antimicrobianos en los registros nacionales de medicamentos y en las listas de medicamentos esenciales de los tres países por medio del Formulario Nacional Británico. También se clasificaron todos los antibióticos según la clasificación de acceso, de vigilancia y de reserva (AWaRe) de la Organización Mundial de la Salud. Se calcularon los porcentajes de los antimicrobianos esenciales y no esenciales que se registraron según la clase de antimicrobianos y la clasificación AWaRe.

**Resultados:**

En 2018, Kenya tenía 2105 antimicrobianos registrados, Uganda 1563 y la República Unida de Tanzania 1327. De estos medicamentos, 1353 (64,3 %) no eran esenciales en Kenya, 798 (51,1 %) en Uganda y 706 (53,2 %) en la República Unida de Tanzania. Kenya tenía 160 antimicrobianos en sus listas nacionales de medicamentos esenciales, Uganda 187 y la República Unida de Tanzania 182; entre ellos, 33 (20,7 %), 50 (26,7 %) y 52 (28,6 %) no estaban registrados, respectivamente. No se registraron altos porcentajes de medicamentos antimicobacterianos y antiparasitarios. De los antibióticos esenciales de acceso, no se registraron el 14,3 % (4/28) en Kenya, el 8,6 % (3/35) en Uganda y el 20,5 % (8/39) en la República Unida de Tanzania, y tampoco se registraron los antibióticos de vigilancia en un 25,0 % (3/12) en Kenya, un 14,3 % (2/14) en Uganda y un 19,1 % (4/21) en la República Unida de Tanzania.

**Conclusión:**

El registro deficiente de los antimicrobianos esenciales y el registro excesivo de los antimicrobianos no esenciales pueden alentar el uso inadecuado, en especial porque los antimicrobianos no esenciales no aparecen en las directrices nacionales de tratamiento. Los países deben dar prioridad al registro de los medicamentos antimicrobianos en sus listas de medicamentos esenciales.

## ملخص

الغرض تحديد نسبة الأدوية المضادة للميكروبات الأساسية وغير الأساسية، والمسجلة في سجلات الأدوية في كينيا وأوغندا وجمهورية تنزانيا المتحدة.

الطريقة قمنا بتصنيف كل مضادات الميكروبات في سجلات الأدوية الوطنية، وقوائم الأدوية الأساسية في الدول الثلاث باستخدام تركيبات الأدوية الوطنية البريطانية. كما قمنا بتصنيف كل مضادات الميكروبات وفقاً لتصنيف الوصول إليها ومراقبتها والحفاظ عليها بواسطة منظمة الصحة العالمية (AWaRe). قمنا بحساب نسب مضادات الميكروبات الأساسية وغير الأساسية التي تم تسجيلها حسب فئة مضادات الميكروبات وتصنيف AWaRe.

النتائج قامت كينيا في عام 2018 بتسجيل 2105 مضاداً للميكروبات، وسجلت أوغندا 1563 مضاداً، وسجلت جمهورية تنزانيا المتحدة 1327 مضاداً. ومن بين هذه الأدوية، كان 1353 مضاداً (64.3%) غير أساسي في كينيا، و798 مضاداً غير أساسي (51.1٪) في أوغندا، و706 مضاداً غير أساسي (53.2٪) في جمهورية تنزانيا المتحدة. النتائج قامت كينيا في عام 160 بتسجيل 2105 مضاداً للميكروبات، وسجلت أوغندا 187 مضاداً، وسجلت جمهورية تنزانيا المتحدة 182 مضاداً. ومن بين هذه الأدوية، كان 33 مضاداً (20.7%) غير أساسي في كينيا، و50 مضاداً غير أساسي (51.1%) في أوغندا، و52 مضاداً غير أساسي (53.2%) في جمهورية تنزانيا المتحدة. وهناك نسب عالية من الأدوية المضادة للبكتيريا والطفيليات، لم يتم تسجيلها. ومن بين المضادات الحيوية ذات الوصول الضروري، لم يتم تسجيل 14.3% (4/28) في كينيا، و8.6% (3/35) في أوغندا، و20.5% (8/39) في جمهورية تنزانيا المتحدة، كما لم يتم تسجيل 20% (3/12) من المضادات الحيوية المراقبة في كينيا، و14.3% (2/14) في أوغندا، و19.1% (4/21) في جمهورية تنزانيا المتحدة.

الاستنتاج إن التسجيل غير الملائم لمضادات الميكروبات الأساسية، والتسجيل الزائد لمضادات الميكروبات غير الأساسية، قد يؤدي إلى تشجيع الاستخدام غير المناسب، وخاصة أن مضادات الميكروبات غير الأساسية لا تظهر في إرشادات العلاج الوطنية. يجب على الدول إعطاء الأولوية لتسجيل الأدوية المضادة للميكروبات في قوائم الأدوية الأساسية لديها.

## 摘要

**目的:**

旨在确定肯尼亚、坦桑尼亚联合共和国和乌干达在药品登记册中登记的基本和非基本抗菌药物的比例。

**方法:**

我们使用《英国国家处方集》(British National Formulary) 对这三个国家的国家药品登记册和基本药物列表中的所有抗菌剂进行了分类。我们还根据世界卫生组织的可用、慎用和备用 (AWaRe) 分类标准对所有抗生素进行了分类。我们计算了按抗菌剂类别以及可用、慎用和备用 (AWaRe) 分类标准登记的基本和非基本抗菌剂的比例。

**结果:**

2018 年，肯尼亚有 2105 种登记的抗菌剂，乌干达有 1563 种，坦桑尼亚联合共和国有 1327 种。在这些药物中，肯尼亚有 1353 种 (64.3%) 非基本药物，乌干达有 798 种 (51.1%) 非基本药物，坦桑尼亚联合共和国有 706 种 (53.2%) 非基本药物。肯尼亚有 160 种抗菌剂列于其国家基本药物列表中，乌干达有 187 种，坦桑尼亚联合共和国有 182 种，其中没有登记的药物分别为 33 (20.7%)、50 (26.7%) 和 52 (28.6%) 种。很高比例的抗分枝杆菌和抗寄生虫药物尚未登记。在必不可少的抗生素中，肯尼亚未登记的比例为 14.3% (4/28)，乌干达未登记 8.6% (3/35)，坦桑尼亚联合共和国未登记 20.5% (8/39)；慎用抗生素未登记的比例在肯尼亚为 25.0% (3/12)，乌干达为 14.3% (2/14)，坦桑尼亚联合共和国为 19.1% (4/21)。

**结论:**

不理想的基本抗菌剂登记和非基本抗菌剂的过度登记可能会导致不当使用，尤其是在非基本抗菌剂未出现在国家治疗指南中的情况下。各国应在其基本药物列表中优先考虑抗菌药物的登记。

## Резюме

**Цель:**

Определить долю основных и неосновных противомикробных препаратов, зарегистрированных в реестрах лекарственных средств в Кении, Объединенной Республике Танзания и Уганде.

**Методы:**

Авторы классифицировали все противомикробные препараты, зарегистрированные в национальных реестрах лекарственных средств и списках основных лекарственных препаратов трех стран с использованием Британского Национального фармакологического справочника. Все антибиотики также были разделены на категории в соответствии с классификацией Всемирной организации здравоохранения для антибиотиков AWaRe (группы доступа, наблюдения и резерва). Авторы рассчитали долю основных и неосновных противомикробных препаратов, которые были зарегистрированы по классу противомикробного действия и классификации AWaRe.

**Результаты:**

В 2018 г. в Кении было зарегистрировано 2105 противомикробных препаратов, в Объединенной Республике Танзания — 1327, а в Уганде — 1563. Из них к неосновным препаратам относились 1353 (64,3%) в Кении, 706 (53,2%) в Объединенной Республике Танзания и 798 (51,1%) в Уганде. В национальном списке основных лекарственных препаратов Кении было 160 противомикробных препаратов, в Объединенной Республике Танзания — 182 и в Уганде — 187, из этих препаратов 33 (20,7%), 52 (28,6%) и 50 (26,7%) соответственно не были зарегистрированы. Значительная часть противотуберкулезных и антипаразитарных препаратов не была зарегистрирована. Что касается антибиотиков, среди основных лекарственных препаратов из группы широкого доступа 14,3% (4/28) не были зарегистрированы в Кении, 20,5% (8/39) в Объединенной Республике Танзания и 8,6% (3/35) в Уганде, а для антибиотиков из группы наблюдения эта доля составила 25,0% (3/12) для Кении, 19,1% (4/21) для Объединенной Республики Танзания и 14,3% (2/14) для Уганды.

**Вывод:**

Недостаточная регистрация основных лекарственных препаратов противомикробного действия и избыточная регистрация неосновных противомикробных препаратов могут способствовать ненадлежащему применению, особенно потому, что неосновные противомикробные препараты не фигурируют в национальных руководящих принципах лечения. Странам следует уделить первоочередное внимание регистрации противомикробных препаратов в списках основных лекарственных препаратов.

## Introduction

The World Health Organization (WHO) promotes the rational use of medicines and universal access to medicines through the WHO Model List of Essential Medicines,^1^ which guides the development of national lists of essential medicines. Essential medicines lists are designed to facilitate the procurement of appropriate medicines and are linked to standard treatment guidelines to ensure the appropriate clinical use of these medicines.^2^ Of the 194 Member States, at least 156 have essential medicines lists.^3^

Although access to essential antimicrobials is fundamental to the human right to health^4^ and a key component of sustainable development goal 3.8,^5^ more than 1 million children in the world die every year from untreated pneumonia and sepsis.^6^ At the same time, antimicrobial resistance is a serious threat to global public health. Increased antibiotic use has mainly occurred in low- and middle-income countries^7^^,^^8^ where improved access to these medicines has outpaced the health systems strengthening needed to ensure their appropriate use.^9^ This increased and often inappropriate use has resulted in a shift towards the use of broad-spectrum and last-resort antibiotics.^8^ In countries of the Organisation for Economic Co-operation and Development, about half of antimicrobial use may be inappropriate.^10^

WHO has warned that “without harmonized and immediate action on a global scale, the world is heading towards a post-antibiotic era in which common infections could once again kill.”^2^ In 2015, the World Health Assembly endorsed a five-point antimicrobial resistance global action plan, a key component of which is optimizing the use of antimicrobials in humans.^2^ In 2017, the WHO Model List of Essential Medicines^1^ adopted the AWaRe classification for antibiotics, that is, access, watch and reserve.^11^ Access antibiotics are those that have activity against common susceptible bacteria and show lower resistance potential than antibiotics in other groups.^11^ Access antibiotics are recommended as first- or second-line treatments and access to these antibiotics should be ensured.^11^ Watch antibiotics have higher resistance potential while reserve antibiotics are the last-resort options that should be reserved for treatment of infections caused by multidrug-resistant bacteria.^11^ WHO recommends that watch and reserve antibiotics be prioritized as targets of national and local stewardship programmes and monitoring.^11^

The East African Community , which comprises Burundi, Kenya, Rwanda, South Sudan, Uganda and United Republic of Tanzania, have a high burden of infectious diseases.^12^ Kenya, Rwanda, Uganda and United Republic of Tanzania have high levels of resistance to common antimicrobials^13^^–^^17^ and suboptimal availability of essential antimicrobials in health-care facilities.^18^^–^^20^ Data on antimicrobial resistance and availability of essential antimicrobials in Burundi and South Sudan are lacking.

Kenya, Uganda and United Republic of Tanzania have national action plans on antimicrobial resistance.^21^^–^^23^ Their essential medicines lists^24^^–^^26^ are linked to standard treatment guidelines^24^^,^^27^^,^^28^ to promote universal access to and appropriate use of antimicrobials. The United Republic of Tanzania has recently adopted the AWaRe classification for antibiotics: access antibiotics should be dispensed at all levels of the health system, watch antibiotics should be dispensed from district hospitals and reserve antibiotics should be used at tertiary hospitals.^24^

Marketing authorization, or registration, by a national medicines regulatory agency is the first step towards making medicines available at the country level. Registrations are typically driven by market potential and may not reflect public health need. For instance, national drug registers include thousands of products, while essential medicines lists in low- and middle-income countries prioritize a few hundred of the most essential medicines. However, the registration of even this smaller subset of essential medicines is not guaranteed if the market is not attractive to pharmaceutical manufacturers. 

Lack of registration of essential medicines may promote the use of non-essential medicines and, in less regulated markets, the use of unlicensed forms of a drug. Medicines circulating on the market without an approval from the national regulatory agency are illegal. Moreover, unregistered medicines may not meet necessary quality standards. Such medicines do not enter the market through reputable channels and are often transported under poor conditions.^29^ The use of unregistered medicines can lead to treatment failure and contribute to antimicrobial resistance if a treatment course contains only a fraction of the correct dose, or the medicine is so badly made that the active ingredients are not dispersed properly.^30^^,^^31^

Systematic studies on registration of essential medicines in sub-Saharan Africa are lacking. A study on registration of first-line drugs for cryptococcal meningitis in low- and middle-income countries showed that neither flucytosine nor amphotericin B was registered in the correct dose and form in Uganda and in the United Republic of Tanzania.^32^ In Kenya, amphotericin B was registered, but flucytosine was not.^32^

Previous analysis of Uganda’s national drug register showed critical under-registration of essential medicines in the country.^33^ Research has shown inappropriate use of medicines when recommended treatment options are not available.^34^

Recently the East African Community harmonized registration processes for medicines and created a common market for medicines within the community.^35^ Therefore, a closer look at registration of medicines in the region is important to understand if non-availability of essential antimicrobials is associated with non-registration. Such information could help policy-makers understand gaps in availability across the region and inform policies to contain antimicrobial resistance, and improve access to essential medicines. 

The aim of our study was to ascertain the extent to which antimicrobials on the essential medicines list and non-essential antimicrobials are registered on the national drug registry in Kenya, Uganda and United Republic of Tanzania. We highlight specific examples of unregistered essential medicines and registered non-essential medicines to show the potential implications for availability and antimicrobial resistance. We could not include Burundi, Rwanda and South Sudan because of the lack of publicly available national drug registers. We determined the registration status of antimicrobials on the national essential medicines lists, which indicates their availability within the country, and calculated the proportion of registered non-essential antimicrobials. We also calculated the proportion of registered and essential antibiotics according to the AWaRe classification. 

## Methods

### Data sources

We used the 2016 essential medicines lists for Kenya and Uganda (688 and 682 essential medicines, respectively) and the 2017 essential medicines list for the United Republic of Tanzania (838 essential medicines) to identify the antimicrobials recommended for use in these countries by their respective ministries of health.^24^^–^^26^

We also accessed the national drug registers for the three countries on 26 February 2018. The national drug registers listed 6151, 3896 and 3590 products for Kenya, Uganda and United Republic of Tanzania, respectively.

### Data extraction

We extracted the following information on antimicrobials from the essential medicines lists: generic name of the medicine, recommended strength and formulation, and the level of the health system at which the medicine should be available and used. We excluded non-medicinal products on the lists as well as veterinary products on Kenya’s national drug register.

We categorized medicines on the essential medicines lists as registered or unregistered according to whether any products with the same generic name, strength and formulation appeared on the national drug registers. We classified medicines on the essential medicines list that were on the national drug register, but in a different strength, route of administration or preparation as unregistered. However, we allowed a different dosage if the different strengths on the national drug register multiplied to the strength recommended on the essential medicines list. We identified and sorted all medicines containing an antimicrobial agent by type of antimicrobial; we sorted antibiotics by class using the British National Formulary system (Box 1).^36^ The antimicrobial combinations category included medicines with two or more different classes of antimicrobial agent. The other category included antimicrobial agents that target more than one class of microorganism. We categorized essential antibiotics as access, watch, reserve or unclassified according to the AWaRe tool from the WHO Model List of Essential Medicines.^1^

Box 1 **Categorization of antimicrobials**

**Antimicrobials**

Antibiotics; antivirals; antifungals; antimalarials; antiparasitics; antimycobacterials; combinations;^a^ and other.^b^

**Antibiotic classes**

Penicillins; macrolides; quinolones and fluoroquinolones; aminoglycosides; first-generation cephalosporins; second-generation cephalosporins; third-generation cephalosporins; fourth-generation cephalosporins; tetracyclines; penems and carbapenems; combinations of different classes of antibiotic; and other antibiotics.

^a^ Contain two or more different antimicrobial agents.^b^ Antimicrobials that target more than one type of microorganism.Note: Categorization was according to the British National Formulary^36^

We calculated the relative proportions of registered and unregistered essential antimicrobials in total and by class. For access, watch and reserve antibiotics that appeared on the essential medicines list, we also calculated the proportion that were not registered.

We categorized medicines on the national drug register as essential or non-essential according to whether their generic name, strength and formulation appeared on the national essential medicines lists. We classified medicines on the drug register that were on the essential medicines lists, but in a different strength, route of administration or preparation as non-essential. We identified all medicines containing an antimicrobial agent from the national drug register. We then categorized antimicrobials and antibiotics using the same system as for the essential medicines lists. We calculated the relative proportions of essential and non-essential antimicrobials and access, watch, reserve and unclassified antibiotics registered in each country.

## Results

For Kenya 20.6% (33/160) of antimicrobials on the national essential medicines lists were not registered, and 64.3% (1353/2105) of the registered antimicrobials were non-essential medicines. For Uganda and the United Republic of Tanzania the proportions for unregistered antimicrobials were 26.7% (50/187) and 28.6% (52/182), respectively and the proportions of non-essential antimicrobials were 51.1% (798/187) and 53.2% (706/1327), respectively (Table 1).

**Table 1:** Antimicrobial medicines on the national drug registers and essential medicines lists, Kenya, Uganda and United Republic of Tanzania, 2018

Information on antimicrobials	Kenya	Uganda	United Republic of Tanzania
Number of antimicrobials on the essential medicines list	160	187	182
Number of unregistered antimicrobials on the essential medicines list (% unregistered)	33 (20.6)	50 (26.7)	52 (28.6)
Number of antimicrobials on the national drug register	2105	1563	1327
Number of non-essential antimicrobials on the national drug register (% non-essential)	1353 (64.3)	798 (51.1)	706 (53.2)

### Unregistered essential antimicrobials

Table 2 shows the proportion of essential antimicrobial medicines that were unregistered. In Kenya and Uganda, antiparasitic antimicrobials had the highest proportion of unregistered essential medicines (35.7%; 5/14 and 57.9%; 11/19, respectively). In both countries, these unregistered essential antiparasitic medicines included pentamidine, suramin sodium and melarsoprol.

**Table 2:** Unregistered antimicrobial medicines on the essential medicines list according to type of antimicrobial and antibiotic class, Kenya, Uganda and United Republic of Tanzania, 2018

Antimicrobial	Kenya			Uganda			United Republic of Tanzania	
	Antimicrobials on list, no.	Unregistered, no. (%)		Antimicrobials on list, no.	Unregistered, no. (%)		Antimicrobials on list, no.	Unregistered, no. (%)
**Antibiotics**								
Macrolides	3	0 (0.0)		3	0 (0.0)		4	0 (0.0)
Penems and carbapenems	2	0 (0.0)		2	0 (0.0)		2	0 (0.0)
Penicillins	10	1 (10.0)		12	1 (8.3)		18	5 (27.8)
Aminoglycosides	4	0 (0.0)		9	2 (22.2)		8	0 (0.0)
First-generation cephalosporins	1	0 (0.0)		1	0 (0.0)		3	0 (0.0)
Second-generation cephalosporins	0	0 (0.0)		2	1 (50.0)		1	0 (0.0)
Third-generation cephalosporins	5	1 (20.0)		2	0 (0.0)		8	2 (25.0)
Fourth-generation cephalosporins	0	0 (0.0)		0	0 (0.0)		1	0 (0.0)
Tetracyclines	1	0 (0.0)		4	0 (0.0)		1	0 (0.0)
Quinolones and fluoroquinolones	5	1 (20.0)		5	0 (0.0)		6	0 (0.0)
Combinations of different classes of antibiotic	6	2 (33.3)		7	2 (28.6)		3	1 (33.3)
Other antibiotics	12	2 (16.7)		15	2 (13.3)		19	7 (36.8)
Subtotal	49	7 (14.3)		62	8 (12.9)		74	16 (21.6)
**Antiparasitics**	14	5 (35.7)		19	11 (57.9)		16	5 (31.3)
**Antivirals**	48	13 (27.1)		42	11 (26.2)		40	11 (27.5)
**Antimalarials**	9	1 (11.1)		12	1 (8.3)		8	2 (25.0)
**Antimycobacterials**	28	7 (25.0)		30	12 (40.0)		25	14 (56.0)
**Antifungals**	9	0 (0.0)		21	7 (33.3)		18	4 (22.2)
**Combinations^a^**	0	0 (0.0)		0	0 (0.0)		0	0 (0.0)
**Other^b^**	2	0 (0.0)		1	0 (0.0)		0	0 (0.0)
**Total**	**159**	**33 (20.6)**		**187**	**50 (26.7)**		**182**	**52 (28.6)**

^a^ Combinations contain two or more different antimicrobial agents.

^b^ Other is antimicrobials that target more than one type of microorganism.

In the United Republic of Tanzania, antimycobacterials had the highest proportion of unregistered essential medicines (56.0%; 14/25). Only one product of a combination of rifampicin, isoniazid, pyrazinamide and ethambutol was registered, and no combinations of rifampicin and isoniazid were registered.

In Kenya, no products of bedaquiline (used to treat multidrug-resistant tuberculosis) were registered. In both Uganda and Kenya, no products containing an intravenous infusion of sulfamethoxazole–trimethoprim in the dose recommended on the respective essential medicines lists were registered.

In total, about a fifth to a quarter of essential antimicrobial medicines were unregistered in the three countries.

### Registered non-essential antimicrobials

Kenya had the highest proportion (69.0%; 864/1253) of registered non-essential antibiotics (Table 3). All second-generation cephalosporins registered in Kenya and almost all those registered in the United Republic of Tanzania were non-essential. All fourth-generation cephalosporins registered in Kenya and Uganda were non-essential. In all three countries, a high proportion of registered combination antimicrobials were non-essential (Table 3). Of these non-essential registered combination antimicrobials, one in Kenya, 13 in Uganda and 14 in the United Republic of Tanzania were topical preparations containing an antibiotic and an antifungal with a corticosteroid.

**Table 3:** Registered non-essential antimicrobial medicines, according to type of antimicrobial and antibiotic class, Kenya, Uganda and United Republic of Tanzania, 2018

Antimicrobial	Kenya			Uganda			United Republic of Tanzania	
	Registered antimicrobials, no.	Registered non-essential antimicrobials, no. (%)		Registered antimicrobials, no.	Registered non-essential antimicrobials, no. (%)		Registered antimicrobials, no.	Registered non-essential products, no. (%)
**Antibiotics**								
Macrolides	152	92 (60.5)		87	49 (56.3)		70	13 (18.6)
Penems and carbapenems	46	26 (56.5		20	9 (45.0)		22	11 (50.0)
Penicillins	318	224 (70.4)		269	180 (66.9)		221	156 (70.6)
Aminoglycosides	64	40 (62.5)		64	33 (51. 6)		38	22 (57. 9)
First-generation cephalosporins	39	36 (92.3)		33	26 (78.8)		30	21 (70.0)
Second-generation cephalosporins	90	90 (100.0)		49	32 (65.3)		40	39 (97.5)
Third-generation cephalosporins	173	98 (56.6)		108	56 (51.9)		91	33 (36.3)
Fourth-generation cephalosporins	13	13 (100.0)		7	7 (100.0)		13	3 (23.1)
Tetracyclines	48	35 (72.9)		26	12 (46.2)		15	10 (66.7)
Quinolones and fluoroquinolones	150	106 (70.7)		114	50 (43.9)		99	46 (46.5)
Combinations of different classes of antibiotic	47	22 (47.8)		54	34 (63.0)		26	17 (65.4)
Other antibiotics	113	82 (72.6)		132	69 (52.3)		95	47 (49.5)
Subtotal	1253	864 (69.0)		963	556 (57.7)		760	418 (55.0)
**Antiparasitics**	152	114 (75.0)		60	22 (36. 7)		56	13 (23.2)
**Antivirals**	193	64 (33.2)		187	68 (36.4)		199	102 (51.3)
**Antimalarials**	133	71 (53.4)		136	49 (36.0)		114	70 (61.4)
**Antimycobacterials**	17	6 (35.3)		25	6 (24.0)		15	1 (66.7)
**Antifungals**	242	157 (64.9)		157	67 (42.7)		137	63 (46.0)
**Combinations^a^**	77	56 (72.7)		28	28 (100.0)		34	34 (100.0)
**Other^b^**	38	21 (55.3)		9	4 (44.4)		12	5 (41. 7)
**Total**	**2105**	**1353 (64.3)**		**1565**	**800 (51.1)**		**1327**	**706 (53.2)**

^a^ Combinations contain two or more different antimicrobial agents.

^b^ Other is antimicrobials that target more than one type of microorganism.

### AWaRe

The highest proportions of registered antibiotics in all three countries were in the access category and the lowest proportion were in the reserve category (Table 4). The proportions of unregistered access antibiotics were 14.3% (4/28) in Kenya, 8.6% (3/35) in Uganda and 20.5% (8/39) in the United Republic of Tanzania. Of the watch antibiotics on national essential medicines lists, 25.0% (3/12), 14.3% (2/14) and 19.0% (4/21) were not registered. In United Republic of Tanzania, two of three reserve antibiotics on the essential medicines list were not registered.

**Table 4:** AWaRe categorization of registered and essential antibiotics, Kenya, Uganda and United Republic of Tanzania, 2018

AWaRe category	Kenya	Uganda	United Republic of Tanzania
**Access**			
Number of registered access antibiotics (% of all registered antibiotics)	503 (40.1)	438 (45.5)	332 (43.7)
Number of essential access antibiotics	28	35	39
Number of unregistered essential access antibiotics (% of all essential access antibiotics)	4 (14.3)	3 (8.6)	8 (20.5)
**Watch**			
Number of registered watch antibiotics (% of all registered antibiotics)	348 (27.8)	279 (29.0)	239 (31.4)
Number of essential watch antibiotics	12	14	21
Number of unregistered essential watch antibiotics (% of all essential watch antibiotics)	3 (25.0)	2 (14.3)	4 (19.1)
**Reserve**			
Number of registered reserve antibiotics (% of all registered antibiotic products)	6 (0.5)	6 (0.6)	3 (0.4)
Number of essential reserve antibiotics	1	0	3
Number of unregistered essential reserve antibiotics (% of all essential reserve antibiotics)	0 (0.0)	0 (0.0)	2 (66.7)
**Unclassified^a^**			
Number of registered unclassified antibiotics (% of all registered antibiotics)	396 (31.6)	240 (24.9)	186 (24.5)
**Total**	**1253**	**963**	**760**

^a^ None of the unclassified antibiotics was on the essential medicines list.

## Discussion

For all three countries, more than half of registered antimicrobials were non-essential and up to a third of essential antimicrobial medicines were not registered.

In the United Republic of Tanzania, the antimycobacterial category had the highest proportion of unregistered essential medicines. Only one product for the combination rifampicin, isoniazid, pyrazinamide and ethambutol was registered, which is recommended for the initial phase of treatment of tuberculosis in standard treatment guidelines.^24^ Furthermore, the country had no registered combinations of rifampicin and isoniazid, which is recommended for continuation therapy in the treatment of newly diagnosed tuberculosis.^24^ The United Republic of Tanzania is on the WHO list of 30 countries with the highest tuberculosis burden in the world and therefore access to appropriate antimycobacterials is vital.^37^

There is multidrug-resistant tuberculosis in Kenya,^37^ but bedaquiline is not registered for use, even though WHO recommends this medicine always be included in the long-course treatment of multidrug-resistant tuberculosis.^38^ The non-registration of some antimycobacterials in Kenya and in the United Republic of Tanzania may be due to parallel tuberculosis programmes that procure medicines internationally and import them on a special import licence.

All three countries have a high proportion of unregistered essential antiparasitic medicines. In Kenya and Uganda, three essential antitrypanosomal medications; pentamidine, suramin sodium and melarsoprol, are not registered. In the early 1960s, trypanosomiasis prevalence fell to low levels in the WHO African region, but a lack of regular surveillance, reduced allocation of resources, changing health priorities and non-availability of medications led to the resurgence of the disease, causing epidemics in some areas.^39^ Therefore, antitrypanosomal medications remain on the essential medicines lists and standard treatment guidelines in both countries.^25^^–^^28^ Although no cases of trypanosomiasis have been reported in Kenya since 2012, and only 10 cases were reported in Uganda in 2016,^40^ the fact that these antitrypanosomal medications are not registered is nevertheless of concern.

Penicillins are categorized as access antibiotics^11^ and are a recommended first-line treatment for several infections in the Tanzanian standard treatment guidelines,^24^ but a high proportion of penicillins in the essential medicines list are not registered. Sulfamethoxazole+trimethoprim is recommended by WHO as the first-line treatment for lower urinary tract infections and second-line treatment for acute invasive diarrhoea and bacterial dysentery.^1^ Although these infections are among the top 10 causes of death in Kenya and Uganda, no products containing an intravenous infusion of sulfamethoxazole–trimethoprim in the dose recommended on the respective essential medicines lists were registered in either country.^41^^,^^42^

Lack of registration of essential antimicrobial medicines risks the use of unregistered products. Availability of unregistered medicines has previously been reported in Kenya and Uganda. In Kenya, 42.2% of antimalaria medicines^43^ and 27.4% (26/95) of antiretroviral medicines^44^ sampled were not registered. A Ugandan study of six tracer medications identified five unregistered brands of rifampicin that were available in medicine outlets.^45^ Such medicines may be substandard. Substandard medicines promote antimicrobial resistance and the spread of drug-resistant infections.^30^ The availability of unregistered medicines is an enforcement issue that is not addressed here.

Expedited regulatory approvals were introduced to facilitate and speed up the registration processes for essential medicines. The WHO collaborative registration procedure allows accelerated registration of finished pharmaceutical products that have been prequalified by WHO,^46^^,^^47^ including products approved through Article 58 of the European Union and the Plan for AIDS Relief of the United States Food and Drug Administration.^48^^,^^49^

Further support for national medicine regulatory agencies comes from regional harmonization of regulatory requirements. The East African Community, in collaboration with the New Partnership for Africa’s Development, launched the East African Community Medicines Registration Harmonization project in 2012.^35^ Joint registrations at this level could also improve access to essential antimicrobial medicines in the region if these products are prioritized.

More than half of the antimicrobial medicines registered in Kenya, Uganda and United Republic of Tanzania are non-essential. Antimicrobial medicines that are not included in essential medicines lists do not appear in standard treatment guidelines, which is likely to lead to the inappropriate use of these non-essential antimicrobials. Our data show that particularly high proportions of non-essential second- and fourth-generation cephalosporins are registered. Second-generation cephalosporins are watch antibiotics, indicating that they have a high resistance potential and their use should be limited. Fourth-generation cephalosporins are reserve antibiotics and as such, should be a last-resort solution for multidrug-resistant infections. Therefore, the high rate of registration of non-essential second- and fourth-generation cephalosporins is alarming.

In addition, we found a high proportion of registered non-essential combination antimicrobials including topical preparations containing an antibiotic and an antifungal with a corticosteroid. A Tanzanian study reported the overprescribing of antibiotics for fungal skin infections and identified the excessive use of fixed-dose combinations of an antibiotic, antifungal medicine and a steroid as a contributory factor.^50^ The registration of such products has the potential to encourage inappropriate use and may consequently contribute to antimicrobial resistance.

Data on the consumption of antimicrobials in Kenya, Uganda and United Republic of Tanzania are still limited. Kenya and Uganda did not submit data to the WHO report on surveillance of antibiotic consumption 2016–2018, while the United Republic of Tanzania submitted only limited import data as a proxy for consumption.^34^ Our findings on the registration status of AWaRe antibiotics support the Tanzanian import data, showing that most antibiotics are in the access category and a significant proportion (over 20%) are unclassified.

Our study has some limitations. We did not examine other legal routes to availability of medicines, including those used by governments and donors, which procure medicines using a special import licence. We accessed the national drug registers for the three countries in February 2018 and the national essential medicine lists valid at that time; we did not take account of any changes since that date.

Alignment of the register of medicinal products and the essential medicines list, which in turn is linked to treatment guidelines, is important to ensure the appropriate use of medicines. The reasons for suboptimal registration of essential medicines in Kenya, Uganda and United Republic of Tanzania need to be examined. All three countries should add explicit commitment to improving drug registration procedures to their national antimicrobial resistance action plans. They should also implement measures to prioritize registration of essential antimicrobials and restrict registration of non-essential antimicrobials. Kenya and Uganda should follow the lead of the United Republic of Tanzania and adopt the AWaRe categorization in their essential medicines lists to increase awareness of resistance potential and the caution needed when access, watch and reserve antibiotics are used.
